# A tool kit for quantifying eukaryotic rRNA gene sequences from human microbiome samples

**DOI:** 10.1186/gb-2012-13-7-r60

**Published:** 2012-07-03

**Authors:** Serena Dollive, Gregory L Peterfreund, Scott Sherrill-Mix, Kyle Bittinger, Rohini Sinha, Christian Hoffmann, Christopher S Nabel, David A Hill, David Artis, Michael A Bachman, Rebecca Custers-Allen, Stephanie Grunberg, Gary D Wu, James D Lewis, Frederic D Bushman

**Affiliations:** 1 Department of Microbiology, Perelman School of Medicine at the University of Pennsylvania, 3610 Hamilton Walk, Philadelphia, PA 19104, USA; 2 Institute for Immunology, Perelman School of Medicine at the University of Pennsylvania, 421 Curie Boulevard, Philadelphia, PA 19104, USA; 3 Department of Pathobiology, School of Veterinary Medicine, University of Pennsylvania, 421 Curie Boulevard, Philadelphia, PA 19104, USA; 4 Department of Pathology and Laboratory Medicine, Perelman School of Medicine at the University of Pennsylvania, 3400 Spruce Street, Philadelphia, 19104, USA; 5 Department of Pathology, University of Michigan, 1301 Catherine St, Ann Arbor, MI 48109, USA; 6 Division of Gastroenterology, Perelman School of Medicine at the University of Pennsylvania, 415 Curie Boulevard, Philadelphia, PA 19104, USA; 7 Center for Clinical Epidemiology and Biostatistics, Perelman School of Medicine at the University of Pennsylvania, 423 Guardian Drive, Philadelphia, PA 19104, USA

## Abstract

Eukaryotic microorganisms are important but understudied components of the human microbiome. Here we present a pipeline for analysis of deep sequencing data on single cell eukaryotes. We designed a new 18S rRNA gene-specific PCR primer set and compared a published rRNA gene internal transcribed spacer (ITS) gene primer set. Amplicons were tested against 24 specimens from defined eukaryotes and eight well-characterized human stool samples. A software pipeline https://sourceforge.net/projects/brocc/ was developed for taxonomic attribution, validated against simulated data, and tested on pyrosequence data. This study provides a well-characterized tool kit for sequence-based enumeration of eukaryotic organisms in human microbiome samples.

## Background

The human microbiome consists of bacteria, archaea, viruses and eukaryotic microbes. Single cell eukaryotes form an important part of these communities, but enumerating community membership and proportions in complex mixtures remains challenging. Advances in sequencing technology and bioinformatics have made possible several strategies. Shotgun metagenomics, in which all DNA from a sample is sequenced, can yield data on the types of organisms and genes present in a mixed community. However, in many types of microbiome samples, eukaryotic microbes are a minor component, so shotgun metagenomics can be inefficient and expensive for their identification. Target gene sequencing can yield detailed information on community membership efficiently, as with the 16S rRNA gene amplicons widely used for profiling bacterial communities. However, there are no universally conserved regions in eukaryotic genomes analogous to those in the 16S rRNA locus of bacteria that yield similarly low level classifications. For microbiome samples from the digestive system, the potential masking effects of food DNA provides another complication, and for many sample types host DNA can also interfere.

Many diseases are mediated by infections of single cell eukaryotes [1-3], including infections of the gut [4], skin [5], urogenital tract [6], and pulmonary system [7]. In some cases infections have been associated with alteration of the normal microbiome [8], as in oral thrush [9] and aspergillosis [10], while others are apparently caused by invasion by a single eukaryotic pathogen such as *Mucor *[10] or *Giardia *[11]. Thus, better understanding of the dynamics of eukaryotic components of microbiome communities will help in understanding and treating many of these infections.

Eukaryotic rRNA genes and their associated transcribed spacers have been used as marker genes [12-15], though target amplicons are not fully universal. In eukaryotes, the 18S, 5.8S, and 28S ribosomal subunits are encoded in a single locus separated by the first and second internal transcribed spacers (ITSs). The ITS RNAs are degraded shortly after transcription and are not incorporated into the ribosome [16]; thus, ITS RNAs are less conserved than the 18S and 28S RNAs. Previously developed eukaryotic rRNA gene amplicons can query these regions, but most have not been designed or vetted for use specifically in human microbiome studies.

Here we describe a pipeline based on rRNA gene amplicons for analysis of eukaryotes of the human microbiome by deep sequencing. Sequencing 18S rRNA genes could be confounded by the potentially more abundant rRNA gene sequences from the mammalian host or, in samples from the gastrointestinal tract, from food. We thus designed an 18S rRNA gene amplicon that avoids mammalian and plant sequences, and also compared a published ITS1 amplicon targeting fungi [14]. We developed a flexible software pipeline (BROCC, for BLAST Read and Operational Taxonomic Unit Consensus Classifier) for attributing sequences that was tailored for use with the complex and sometimes inconsistent taxonomic assignments characteristic of single cell eukaryotes. Because some fungi can be hard to lyse, we compared four methods for lysis and DNA purification. Performance was tested over 24 DNA samples from known eukaryotes and eight human stool samples. No single marker gene strategy can quantify all eukaryotic sequences in a sample, but the methods described here allow characterization of a large and well-characterized subset.

## Results

### DNA from food is detectable in fecal material

Humans consume other eukaryotes as food, so in order to design maximally useful amplicons for the detection of eukaryotic rRNA gene sequences in gut microbiome samples, we first investigated the survival of DNA during passage through the gut. In an early study of this issue, plasmid DNA was fed to mice and low molecular weight DNA from pellets was found to contain apparent plasmid-derived DNA, which was detected as smears on Southern blots [17]. Another study showed that 16S rRNA gene sequences in pellets of gnotobiotic (germ-free) mice resembled 16S sequences in mouse food [18]. Our own evidence from shotgun metagenomic studies also suggested that DNA from food may be detectable in human stool [19], though this has not been studied in detail. In a further study (data not shown), we gavaged mice with purified bacterial plasmid DNA and showed that plasmid DNA could be detected in fecal pellets 6 hours but not 60 hours after feeding using Taqman Q-PCR. Based on these observations, we sought to identify eukaryotic rRNA gene amplicons that could detect single cell eukaryotes of the human microbiome while selectively avoiding amplifying rRNA genes from food organisms and host.

### Design of amplicons

**Figure 1:**
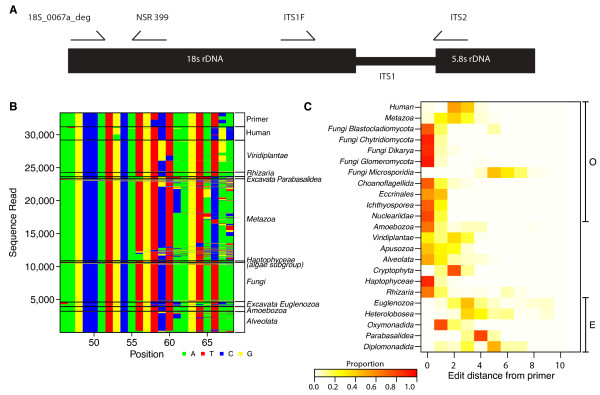
**The eukaryotic ribosomal DNA locus and the targets of amplicons studied here**. **(a) **Part of the rRNA gene locus showing primer binding sites (not to scale). **(b) **Comparison of sequence complementarity for the 18S-0067a-deg primer against various eukaryotic groups. Clades belonging to Excavate (E) or Opisthokont (O) groups [27,41] are marked on the left. Apusozoa consist of ciliated protozoans [42]. Cryptophyta and Haptophyceae are subgroups of algae [27]. **(c) **Heat map indicating the edit distance (numbers of mismatches) between the 18S-0067a-deg primer and target sites in the indicated organisms. Colors are scaled to the relative proportion of each taxa represented at each edit distance.

We targeted the 18S rRNA gene (Figure 1a) due to its high conservation among eukaryotes [20] and the substantial bioinformatic resources available for 18S rRNA gene analysis [21,22]. We analyzed 18S rRNA gene sequences from the Silva database [21] and manually scanned alignments for mammalian- and plant-specific polymorphisms. A primer was designed (18S_0067a_deg; Figure 1b, c) that showed low edit distance (high identity) to 18S rRNA genes of fungi, Amoebozoa, chromalveolates, Rhizaria, and most excavates, but showed lower identity to human 18S rRNA genes due to mismatches at the 3' end. In addition, some though not all plants showed relatively high edit distance to 18S_0067a_deg (Figure 1b, c). We paired it with the universal NSR399 18S rRNA gene primer, which is complementary to all eukaryotic clades [23].

The 18S rRNA gene is not sufficiently polymorphic for classification of some groups at a low taxonomic level [15], so we also tested an ITS1 primer set, which queries a less-conserved region and targets fungi selectively. We used a version of the ITS1F/ITS2 primer set previously reported to show discrimination at low levels of the fungal taxonomy [14].

All primers used for amplification also contained a DNA bar code, which consisted of 12 bases that indexed the DNA specimen studied. Sequence reads could then be separated by bar code during bioinformatic analysis, allowing many amplicons to be sequenced in pools.

### Classification of amplicon sequences using BROCC

Classifying sequences from microeukaryotes presents special challenges in automated assignment: first, there are large numbers of accepted synonyms for many taxonomic groups; second, databases contain an unusually high level of misclassifications; third, sexual and asexual forms (anamorphs and telomorphs) of a single fungal species can be in different taxa, even up to the family level; and fourth, databases contain large numbers of environmental sequences with minimal or no classification that nevertheless are returned as hits from database searches. For these reasons, we designed BROCC to classify single cell eukaryotes while respecting these limitations. BROCC also facilitates interfacing with the popular QIIME pipeline [24], which was originally developed for use with bacterial 16S rRNA gene tags.

We chose to use a BLAST-based method, rather than a kmer-based classifier such as RDP [25], because the high level of variation between closely related ITS sequences could result in misplaced assignments. Phylogenetic-based methods such as ARB [22] have difficulties with ITS sequences because of rapid divergence and common indels.

BROCC classifies amplicons using BLAST searches against large and relatively uncurated databases. There are curated databases for several eukaryotic amplicons that can be used for phylogenetic assignment [15,21], but large curated databases do not exist for ITS1, which is used here. It is widely speculated that the great majority of fungi have not been studied, motivating use of the broadest possible databases for human microbiome studies. BROCC uses blastn, but output from other versions of BLAST, such as blastx, can be substituted. Parameters are user-adjustable. BROCC first filters input BLAST hits for sufficient coverage and identity to the query sequence. If a query sequence has too many hits that are below the preset coverage threshold (70% default), or BLAST did not return a hit, it is not classified, and a message is written to the output file. BROCC then determines the identity and taxonomic hierarchy of each high quality hit using a local user-installed sql database and the NCBI's e-fetch tool.

BROCC then votes on the quality filtered BLAST hits, starting at the species level. At each level of the taxonomy BROCC requires the taxon with the most votes to surpass a user-specified threshold for that level in order to accept it as a valid classification. If a sufficient majority is not reached, BROCC will not make a classification for that level and iterate to the next higher taxonomic level for another round of voting. BROCC filters are independently configurable at the genus and species levels, and another filter can be assigned for the remaining taxonomic levels. Here different defaults were used for ITS and 18S rRNA gene amplicons. Species and genus defaults for ITS rRNA gene amplicons were chosen on the basis of [26], and are 95.2% and 83.05%; 80% was used for higher taxa. For 18S rRNA gene amplicons, experience (data not shown) indicated that 99% was suitable for species attribution, 96% for genus, and 80% for higher levels.

BROCC also contains a user-modifiable list of high level and partial assignments in its configuration file. These assignments are ignored at lower taxonomic levels where they are uninformative and can distort voting, but included in higher levels. For example, a sequence read with a kingdom level assignment only is excluded up to the kingdom level, at which point the vote is counted in the kingdom assignment. In cases where the proportion of high level and partial assignments exceeds a given threshold (default 0.70), the query sequence is unassigned and marked accordingly.

BROCC output includes both files containing classifications with standardized taxonomy (domain, kingdom, phylum, class, order, family, genus, species) and a second with the complete NCBI taxonomy [27], which includes subtaxa, supertaxa, and unranked intermediate taxonomic levels. The third file contains a log of the voting record, including how many votes were cast, how many votes the winning taxon received, and how many generic classifications were ignored for each query sequence. This file also indicates those queries that were unclassified. Both taxonomy files are suitable for use in the QIIME pipeline (that is, they are in the same format as the output classifications as the QIIME assign_taxonomy.py script).

### Testing BROCC performance on an *in silico*-constructed community of known membership

**Figure 2:**
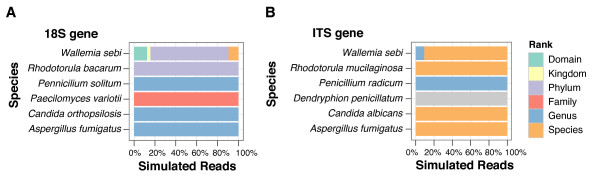
**Classification of *in silico*-generated mixed communities of eukaryotic rRNA gene sequences using BROCC**. For each community, sequences from six different organisms were pooled, with 32 sequences per organism. Sequences contained 1% added error and variable length truncations. The organism chosen is shown to the left of each panel, and assignments are shown to the right of each figure panel by the color code. **(a) **18S rRNA gene assignments. **(b) **ITS rRNA gene assignments.

We next verified performance of BROCC by testing assignments over an *in silico*-generated mixed community of known membership (Figure 2). We selected six eukaryotic microbial organisms, and extracted sequences corresponding to our 18S and ITS rRNA gene amplicon regions. To simulate the characteristics of pyrosequencing data, we added base substitution errors at a rate of 1% and truncated each sequence by a length selected randomly from an exponential distribution, such that the average trim value was five bases. For each strain, 32 different reads were generated, and then classified by BROCC.

For the 18S rRNA gene sequences, most reads were classified to at least the genus level for four of the six organisms. One of the remaining two was classified at the family level, and another was classified at only the phylum level (*Rhodotorula bacarum*). For *Rhodotorula*, the NCBI taxonomy jumps from phylum to genus, disrupting attribution. For the ITS amplicon, four of the six organisms were classified to the species level and one was classified at the genus level (*Penicillium*). *Dendryphion *was unclassified, due to an abundance of short sequence matches in the database that covered less than 70% of the ITS query and thereby disrupted assignment. We conclude from this that 1) BROCC works well for attribution even in the presence of sequence errors and truncations, 2) the ITS amplicon yields lower level assignments than the 18S rRNA gene amplicon for those sequences accessible with the ITS primers used, and 3) failed assignments were mainly attributable to problems in the underlying database.

### Testing the pipeline using a collection of DNAs from microeukaryotes of clinical interest

**Figure 3:**
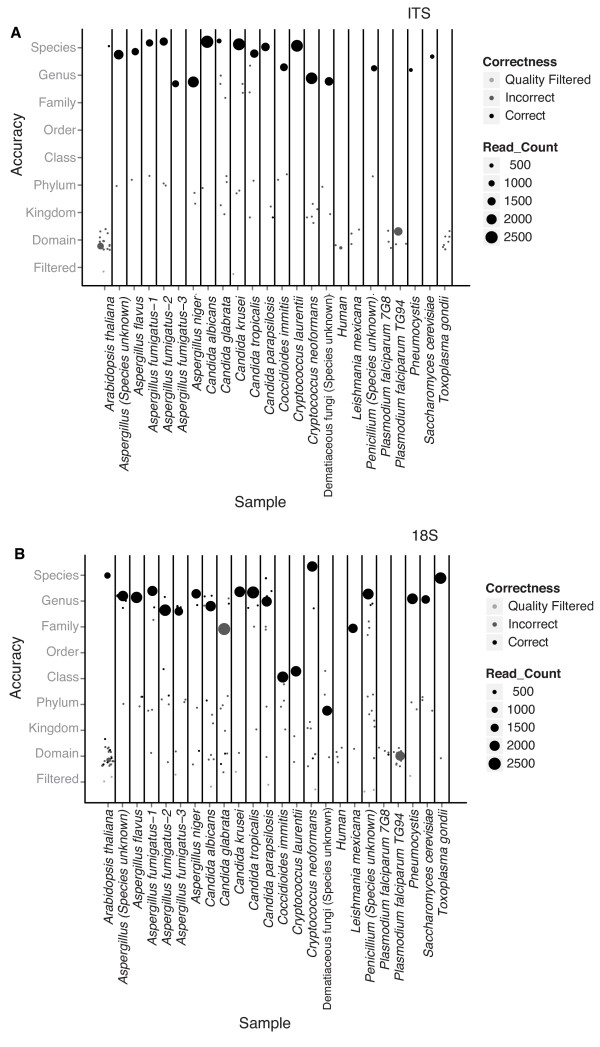
**Analysis of DNA samples from known eukaryotes**. **(a) **18S rRNA gene amplicons. **(b) **ITS rRNA gene amplicons. The sample tested is listed along the x-axis. The y-axis shows the level of taxonomic placement of each OTU in each sample relative to the correct taxon indicated on the x-axis. The numbers of sequence reads are shown by the size of the point. Thus, large circles high up on the y-axis indicate correct placement of the major taxa.

In order to test the performance of our pipeline, we tested DNA extracted from clinical isolates of fungi and molds, as well as selected laboratory strains of model eukaryotes (Additional file 1). We also tested DNA from humans and *Arabidopsis thaliana*, which are selectively non-targeted organisms. DNA samples were amplified with our 18S and ITS rRNA gene primer pairs and sequenced using the 454/Roche platform. The raw sequences (54,698 for 18S rRNA genes, 35,259 for ITS genes) were processed and denoised in the QIIME pipeline [24]. Operational taxonomic units (OTUs) were formed with percent identity values used for species-level attribution above. Taxa were assigned using BROCC. We scored a BROCC classification as correct if it returned an accepted synonym, anamorph, or teleomorph from the Mycobank database [28] or the NCBI taxonomy database matching the known assignment (Figure 3a, b).

For the 18S rRNA gene amplicon, of the 23 classified samples tested, the major OTU was annotated as the correct organism at the family level or lower for 18 specimens and at the genus level or lower for 16 specimens (Figure 3a). Taxa called correctly at the genus level or lower included *Aspergillus*, most Saccharomycetaceae yeasts (*Candida *and *Saccharomyces*), *Penicillium*, *Pneumocystis*, and *Toxoplasma gondii*. A low number of reads were detected for *A. thaliana *despite the effort to avoid amplifying plant DNA, and these reads were also correctly placed. Taxa called correctly to the family level included *Leishmania *and *Candida glabrata*. *Coccidioides *was called correctly at the class level. The human DNA sample yielded only low numbers of reads, and the most abundant OTU matched Saccharomycetaceae yeasts, consistent with the idea that only low level contaminating environmental DNA amplified from these samples. *Plasmodium *did not amplify with this primer pair, consistent with the large edit distance between the primer sequences and the rRNA gene target.

*Cryptococcus neoformans *classified correctly to the species level, but *Cryptococcus laurentii *initially only classified correctly to the phylum level. Analysis showed this was due to a large number of database entries for closely related sequences annotated as 'Uncultured soil basidiomycete'. We thus added this term to our list in BROCC of unhelpful classifications to be excluded, after which *C. laurentii *was correctly classified to the class level.

For the ITS gene amplicon, of the 23 samples tested, the major OTU was annotated as the correct organism at the genus level or lower for 18 specimens (Figure 3b). Taxa called correctly included *Aspergillus*, *Cryptococcus*, *Penicillium*, *Pneumocystis *and Saccharomycetaceae yeasts (*Candida *and *Saccharomyces*). Human and *Arabidopsis *were not correctly called and the major OTU corresponded to a *Saccharomyces *yeast, consistent with low level contamination. We also failed to correctly call the apicomplexan samples (*Plasmodium *and *Toxoplasma*), consistent with the presence of several mismatched base pairs in the forward primer, and *Leshmania*. Again, for most of these the numbers of reads were low and corresponded to abundant environmental fungi that were probable contaminants.

One clinical strain was dubbed a dematiaceous mold, which is not a taxonomic identifier. Analysis of the 18S rRNA gene amplicon data called it only as Ascomycota, because divergent annotation at lower levels obstructed deeper classification by BROCC. However, analysis of the ITS amplicon data called it as genus *Exophalia*, which fits with the clinical profile.

Most samples also showed additional low level OTUs, usually represented by less than five sequence reads unrelated to the correct call. In some cases these were identifiable as common environmental fungi that likely contaminated either the original DNA samples or reagents used for DNA purification. Extensive amplification of extraction negative controls occasionally yielded such OTUs (data shown below). Other low level OTUs in Figure 3 were not identified and may be products of mispriming, chimera formation, or pyrosequencing error.

### Comparison of DNA purification methods

Choice of cell lysis and DNA extraction methods influences both the DNA yield and proportions of taxa for bacterial 16S rRNA gene analysis [29], and the known difficulties of lysing yeasts suggest the issue may be even more pronounced here. We thus compared four different extraction methods for preparing samples for analysis of eukaryotic rRNA gene sequences: PSP Spin Stool DNA Plus Kit, MoBio PowerSoil kit, FastDNA with Fungal Protocol [14], and an archaeal-specific extraction method [30]. For some, harsher lysis steps were used than in the original protocols (see Materials and methods). Eight stool samples from healthy adults were subjected to separate extractions with each of the four kits. The PSP kit yielded the most DNA on average for the same weight of starting material. Output DNA from each method was then tested using both the ITS1 and 18S rRNA gene amplicons.

Amplification products were separated by agarose gel electrophoresis and visualized by staining with ethidium bromide (Additional file 2). The genomic DNA from the FastDNA protocol produced no detectable amplification. The PSP and PowerSoil extractions produced similar banding patterns on ethidium bromide-stained agarose gels, though the PSP extractions produced brighter bands overall. The archaeal extraction method produced sporadic bands that were generally less bright than the PSP and PowerSoil samples. Based on these findings, the PSP kit seems superior. The archaeal, PSP, and PowerSoil samples were then compared after deep sequencing by the 454/Roche method.

### Comparison of taxa reported with the 18S and the ITS rRNA gene amplicons for human stool samples

**Figure 4:**
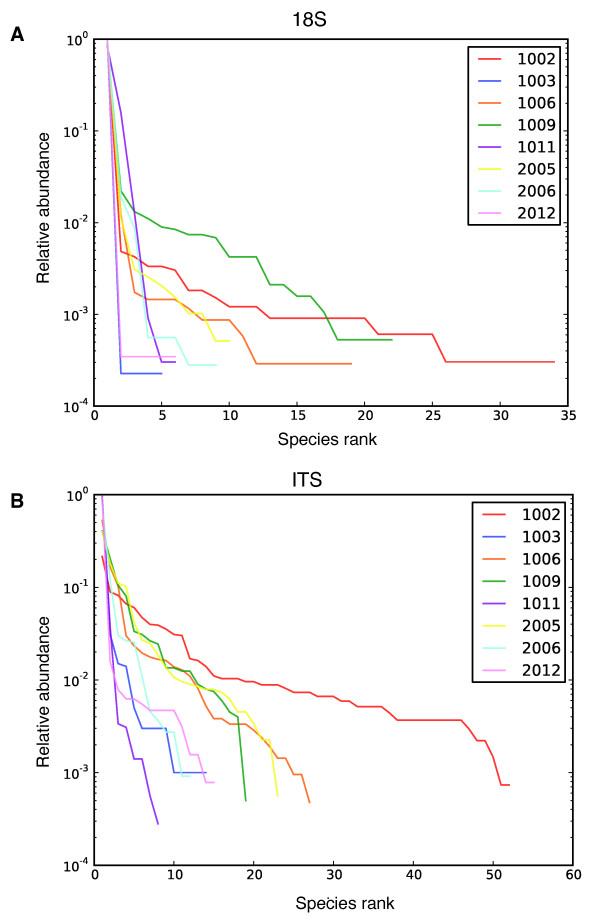
**Rank-abundance plots for operational taxonomic units from stool samples**. **(a) **18S rRNA gene amplicons. **(b) **ITS rRNA gene amplicons. The rank (relative abundance) of each OTU is shown on the x-axis, with the most abundant on the left. The proportion contributed by that OTU is shown on the y-axis. The key in the upper right shows the color code for the different human subjects studied.

We acquired 54,411 sequence reads for the 18S rRNA gene amplicon and 39,827 sequence reads for the ITS1 amplicon from the 8 stool samples (Additional file 3). The sequence reads were clustered into OTUs and assigned to eukaryotic taxa using BROCC. The relative abundance of community members was assessed by plotting OTUs ranked by abundance versus their within sample abundance for samples extracted with the PSP method (Figure 4a, b). The 18S rRNA gene amplicon yielded 93 OTUs and the ITS amplicon yielded 215 OTUs. For both the 18S and ITS rRNA gene amplicons, a few OTUs contained most reads, and this was more pronounced for the 18S rRNA gene amplicon data. The majority of OTUs assigned by BROCC from both amplicons belonged to fungal phyla (62.4% in 18S and 90.5% in ITS1 rRNA gene amplicons), mainly Ascomycota (81.0% in 18S and 57.4% in ITS1 rRNA gene amplicons) and Basidiomycota (17.2% in 18S and 25.7% in ITS1 rRNA gene amplicons). Recovery of plant and animal DNA from the 18S and ITS rRNA gene amplicons was suppressed effectively. Only two OTUs in the 18S rRNA gene amplicon totaling 35 reads and 5 OTUs in the ITS amplicon totaling 5 reads were classified as plant. No OTUs were classified as vertebrate, though in other experiments with these primers small numbers of host and vertebrate sequences have been detected (data not shown).

**Figure 5:**
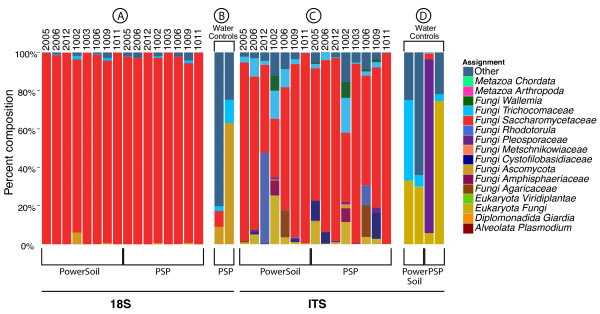
**Comparison of major eukaryotic microbes detected in human stool**. Samples were assayed with the 18S rRNA gene amplicon, the ITS1 rRNA gene amplicon, and the shotgun genomic data in human stool. Human subjects and DNA purification methods are as indicated on the x-axis. Taxa are shown at the family level or as indicated. **(a) **18S rRNA gene amplicon used to analyze stool samples. **(b) **18S rRNA gene amplicon contamination controls. **(c) **ITS amplicon stool samples. **(d) **ITS rRNA gene amplicon contamination controls. The contamination controls in (b, d) consisted of DNA-free water passed through the full DNA purification, sequencing and analytical pipeline; six of eight samples yielded pyrosequence data, though with low read numbers.

The numbers of reads returned for each OTU can be used as a surrogate for relative abundance, though this measure must be used with caution due to unequal amplification due to internal secondary structure, differential complementarity of target sequences and primers, and different amplicon lengths. The proportions of sequences are shown as stacked bar graphs in Figure 5 for the PSP and PowerSoil extraction methods. Yields from the archaeal extraction were lowest of the three, and showed multiple samples with few or no reads, and so were not studied further. Sequence reads were detected in six of eight negative controls (Figure 5b, d), in which DNA-free water was subjected to the purification, amplification and sequencing procedures, but the read numbers were typically much lower than for the stool samples (Additional file 3).

For the 18S rRNA gene amplicon, 99.6% of fungal reads were assigned to Ascomycota, while the rest were assigned to Basidiomycota, except for a three-read OTU assigned to *Entomophthora*. For the ITS amplicon, 83.7% of fungal reads were assigned to Ascomycota, 9.79% were assigned to Basidiomycota, and 6.4% were only classified to the kingdom level. Twelve reads from PowerSoil extraction of subject 1006 were assigned to Mucoromycotina.

The 18S rRNA gene amplicon also detected two gut parasites, *Blastocystis *and *Endolimax*. These were not detected using the ITS amplicon, which is specific for fungi. The DNA extraction method used affected the results - *Blastocystis *was detected in both the PSP and PowerSoil extractions from subject 2006 and *Endolimax *in the PSP extraction in subject 2006, but not in samples extracted by other methods. It is unclear whether this divergence is due to bias in the extraction methods or uneven distributions of organisms in stool samples.

The Saccharomycetaceae proved to be the dominant lineage in the eight stool samples for both the 18S and ITS1 rRNA gene amplicons. Both amplicons were dominated by *Saccharomyces *and *Candida *genera (Figure 5a, c). Most Saccharomycetaceae reads recovered with the 18S rRNA gene amplicon were classified as *Saccharomyces *in all samples. However, for the ITS1 rRNA gene amplicon, reads were classified as a mixture of *Candida *and *Saccharomyces*. Analysis of the 18S rRNA gene sequence over the window queried by our amplicon revealed that *Saccharomyces *and *Candida *are poorly distinguished over this region, which was corroborated by a multilocus phylogeny over the Saccharomycetaceae family [31].

Aside from the typical gut inhabitants, our study yielded several examples of fungal rRNA genes potentially derived from food. In subject 1006, *Agaricus bisporus*, the common button mushroom, was detected as a high count OTU using all extraction methods for the ITS1 amplicon samples. *Claviceps purpurea*, which grows on rye and other cereals and is a causative agent of ergot [32], was detected as a rare OTUs in subjects 1002, 1006, and 2006. *Wallemia sebi*, often found in food [33], was detected in 1002, 1006, 1009, and 2005 for multiple extraction methods. The substantial amount of *Saccharomyces *that appeared in all subjects may be derived from bread, beer, or other leavened and fermented foodstuffs. Distinguishing fungal sequences derived from food presents an ongoing challenge in gut microbiome studies.

### Comparison of the performance of BROCC to other classifiers over the experimental data sets

Taking advantage of these data, we next compared BROCC to two other classifiers, MEGAN and MARTA, which were not specifically designed for use with single cell eukaryotes (Additional file 4). Additional file 5 summarizes the differences among the programs. For more discussion of the assignment problem, see [34-37] and references therein.

The three programs were first tested by comparing the number of correct assignments for the known samples in Figure 3. The number of samples with correct assignments for the 18S rRNA gene amplicon at the genus level or lower was 17 for BROCC, 19 for MARTA, and 3 for MEGAN out of 20 possible. For the ITS amplicon, the numbers were 18 for BROCC, 11 for MARTA, and 6 for MEGAN out of 18 possible. Thus, BROCC and MARTA were comparable, with BROCC performing somewhat better for the ITS gene amplicon. MEGAN was more conservative and made fewer low level assignments for ITS, because it was more strongly influenced by database errors or alignments with only high level taxonomic placements.

In some comparisons, MARTA yielded more low level classifications due to accepting single high quality matches for assignment, which can be an advantage or disadvantage depending on the quality of the underlying database. MARTA classified *Candida krusei *as *Pichia fermentans *in the 18S rRNA gene amplicon and *Coccidioides immitis *as *Coccidioides posadasii *in the ITS amplicon. MARTA considered 4 database hits for *C. krusei *and 6 for *C. immitis*, while BROCC considered 98 for *C. krusei *and 27 for *C. immitis*. In both cases BROCC made a correct genus level assignment only and not the erroneous species level assignment. In four cases in the ITS amplicon assignments, MARTA failed to make an assignment due to interference from multiple aligning database sequences assigned as 'unidentified' or 'uncultured', which were correctly classified to low taxonomic levels by BROCC.

We then compared the assignments for BROCC and MARTA against the human stool samples, for which the composition is not known. MEGAN was not considered further due to inferior performance on the known samples. We assigned each classification level a score. Species level assignments received value 1, genus value 2, and so on up to unclassified, which received value 9. Scores were compared between BROCC and MARTA. This showed that BROCC consistently yielded lower level classifications (Wilcoxon signed rank test; *P *= 0.014 for the 18S rRNA gene amplicon, and *P *= 4.1 × 10^-15 ^for the ITS amplicon). Inspection of the data showed the numbers of unclassified OTUs generated by MARTA was largely responsible for the inferior score.

BROCC also contains functionality assisting in implementation that is lacking in the other packages (Additional file 5). BROCC can extract useful information from partial assignments - for example, a database hit assigned only at the kingdom level is not tallied during the process of assignment at lower ranks, but considered in the case of a kingdom assignment. BROCC reports the reason for excluding database hits in the output file. BROCC also outputs file types that are easily integrated into the QIIME pipeline [24] for evaluation of microbial community structure, accelerating downstream steps in a typical analysis.

## Discussion

Here we present a pipeline for characterization of eukaryotic taxa in microbiome samples. For many types of samples, single cell eukaryotes are a minority component, so that shotgun metagenomic analysis is inefficient and expensive. Thus, despite the rapid advance of methods, marker gene analysis remains the method of choice for many applications.

We describe experiments to characterize the performance of two primer sets querying the eukaryotic ribosomal rRNA genes. Data from us and others show that interfering DNA from food or host cells must be considered in designing the amplification strategy. We thus devised an 18S rRNA gene amplicon that selectively avoids plant and animal 18S rRNA gene sequences. We also studied a second amplicon that targets ITS sequences from fungi, which also minimizes contamination with plant and animal DNA but queries a narrower group of eukaryotes. The ITS rRNA gene region studied is more diverse than the 18S rRNA gene region, allowing lower level phylogenetic placement of some fungal groups. Both amplicons were effective in detecting *Aspergillus*, Saccharomycetaceae, *Penicillium*, and *Pneumocystis*. The 18S rRNA gene amplicon selectively detected *Leishmania *and *Toxoplasma*. In stool, the 18S rRNA gene amplicon but not ITS detected *Blastocystis *and *Endolimax*. The ITS amplicon selectively classified *Cryptococcus *and the dematiaceous mold. Neither primer set detected *Plasmodium*. Both amplicons detected Saccharomycetaceae yeast as the major group in stool samples. In unpublished work, the ITS amplicon has also been used to characterize bronchoalveolar lavage samples that were also typed in clinical culture-based assays, producing nearly identical assignments (E Charlson, R Collman, and FDB, unpublished data).

The present state of fungal taxonomy creates challenges in data analysis. Most fungi have not yet been formally described by taxonomists [38], so many sequence reads will be from unknown groups. Names differ for anamorphs (asexual forms) and teleomorphs (sexual reproductive forms) of what are apparently the same species, either of which may occur in the microbiome [39]. Consequently, several OTUs were classified with different names, but belonged to the same holomorph (pool of anamorphs and teleomorphs). Even though they are the same holomorph, *Candida *is taxonomically placed in the family Saccharomycetaceae, but *Clavispora *is placed in the family Metschnikowiaceae. Efforts to improve databases by eliminating the dual naming system and creating accurate phylogenies for fungi should help in this regard [15].

We demonstrated that DNA can survive passage through the gastrointestinal tract of a mouse, albeit inefficiently, and our rRNA gene amplicon assays of human stool did detect some OTUs that likely came from food. For some of the fungal groups, it is difficult to know whether they are true gut residents or transients from food. Perhaps the development of detailed databases of eukaryotic rRNA gene sequences common in human food can assist in distinguishing true gut residents from transients.

## Conclusions

We have described a pipeline for assessing the eukaryotic component of the human microbiome, which includes tested DNA isolation methods, amplification primers targeting the eukaryotic rRNA locus, and software for attribution. Applications for these methods must be chosen with some care - sequences from different species can be recovered with different efficiencies, and frequencies will differ between the 18S and ITS rRNA gene amplicons. Comparison of communities to each other using a single amplicon works well to identify clustering or gradients associated with environmental variables, because amplicon-specific effects are common among all samples. Other applications can be more problematic. The relative abundance of taxa within a sample may be distorted due to differential recovery of different length molecules or interfering secondary structure. Attribution of sequences at low taxonomic levels can be uncertain. Despite our optimization of DNA recovery methods, it remains likely that hard-to-lyse cells and spores are under-represented. Thus, the methods described here are best used for 1) comparing among communities, 2) providing an overview of eukaryotic lineages in a community at a relatively high taxonomic level, and 3) generating hypotheses for specific species present.

## Materials and methods

### Sample collection

Isolates of *Aspergillus*, *Candida*, *Penicillium*, *Cryptococcus*, and dematiaceous mold were obtained from the Clinical Microbiology Laboratory at the Hospital of the University of Pennsylvania. Cultures were treated at 95°C for five minutes to sterilize before removal from the laboratory. The *Pneumocystis*, *Coccidioides*, *Leishmania*, *Toxoplasma*, *Plasmodium*, *Arabidopsis*, *Saccharomyces *and human samples were from lab strains at the University of Pennsylvania. The samples were bead-beaten for 1 minute, heat inactivated for 5 minutes at 95°C and then DNA was extracted with the QIAamp DNA Stool Mini Kit (Qiagen Sciences, Germantown, MD, USA) using the manufacturer's protocol. In subsequent studies we have found that the QIAamp DNA Stool Mini Kit is not DNA free (data not shown), explaining the origin of some of the background sequences. The human stool samples were from healthy adults described in [19,29].

### Primer design

The 18S_0067a_deg primer was designed by screening a set of aligned eukaryotic 18S rRNA gene sequences downloaded from the Silva database [21] and searching for mammal-specific polymorphisms in the 5' conserved regions that flank the hypervariable regions. Three bases at 65-67 were conserved in nearly all 18S rRNA genes but were absent in mammalian 18S rRNA genes, providing the basis for designing selective primers. The NSR399 primer was obtained from the European Ribosomal RNA Database. The ITS amplicons were amplified with the ITS1F/ITS2 primers as in Ghannoum *et al. *[14]. Sequences are given in Additional file 6.

### DNA purification

DNA was purified from human stool (stored frozen at -80°C) using four different methods as specified by the manufacturer except where noted. Approximately 220 mg of stool was used for each extraction.

The FastDNA extractions were done with the FastDNA kit as described by Ghannoum *et al. *[14], except the FastPrep Instrument was replaced by a Mini-Beadbeater-16 (BioSpec Products, Bartlesville, OK, USA). The archaeal extractions were preformed according to the methods of Dridi *et al. *[30]. The PowerSoil extractions were bead beaten for 1.5 minutes in MoBio garnet tubes and centrifuged at 1,500 rcf for 5 minutes. Supernatant (1 ml) was transferred to a PowerBead Tube and heated at 65°C for 10 minutes and then 95°C for 10 minutes. We then used the manufacturer's protocol, skipping the first sample vortex (steps 1 and 2) and spun for 2 minutes instead of 1 at the spin filter loading step (step 15). The samples that were purified with the PSP extraction method were placed in Lysing Matrix E tubes (MP Biomedicals, Solon, OH, USA) with 1,400 μl of stool stabilizer from the PSP kit and were bead beaten in a Mini-BeadBeater-16 (BioSpec). Samples were then heated at 95°C for 15 minutes, placed on ice for 1 minute, and spun down at 13,400 g for 1 minute. The supernatant was then transferred to the PSP InviAdsorb tubes and the rest of the protocol for the PSP Spin Stool DNA Plus was followed according to the manufacturer's instructions. As controls, DNA free water was passed through each DNA extraction procedure, amplified, and samples were sequenced even in cases where no DNA was detectable after amplification ('water controls' in Figure 5).

### Sequence acquisition

Primers with 12 base barcodes were used for 454 FLX sequencing. DNA was initially amplified with AccuPrime DNA polymerase and buffer 2 (Invitrogen Carlsbad, CA, USA). The PCR was carried out with a 5 minute denaturing step at 95°C, followed by 35 cycles of a 45 s denaturing step at 95°C, a 45 s annealing step at 56°C, and a 1.5 minute extension step at 72°C. Finally, there was a 10 minute extension step at 72°C and samples were held at 4°C. The resulting amplicons were then sequenced on a Roche 454 Junior instrument using the FLX Titanium chemistry according the manufacturer's instructions.

### Bioinformatic analysis

Raw sequence data were denoised and analyzed using the QIIME pipeline [24]. OTUs were formed by CD-HIT [40] at 99% convergence for the 18S rRNA gene amplicon and 95.2% convergence for the ITS1 amplicon. The last 20 bases in reads from the 18S rRNA gene amplicons were trimmed due to low overall quality. Homopolymer limits in the read quality filtering were disabled for the ITS1 amplicon.

### The BROCC classifier

BROCC classifies query sequences by voting on BLAST hits scored by identity. All hits are filtered for identity and coverage. Classifications are voted on in a bottom up fashion, starting at the species level. Specific identity filters are specified by the user for the genus and species levels in addition to the main identity filter used for all other levels. Once a classification is made at a given level, all the higher levels are called automatically. If a consensus is not reached at a given level, that level and lower levels are left blank in the final classification. Genus and species identity filters were set at 83.05% and 95.2% for the ITS1 amplicon and 96% and 99% for the 18S rRNA gene amplicon. All other levels were filtered at 80%. The minimum coverage and generic classification filters were set at 70% for all amplicons. Classifications at the species through family levels required a 60% majority to be accepted. Classifications at the order level and above required a 90% majority to be accepted. Pseudocode and a graphical description of BROCC are provided in Additional file 7. The BROCC program is implemented in Python version 2.7. It queries the NCBI taxonomy and requires local installations of SQL and BLAST. The online BLAST user interface was used in error checking. BROCC parameters used are listed in Additional file 8. Source code for BROCC version 1.1.0 is located in Additional file 9.

## Abbreviations

BROCC: BLAST Read and Operational Taxonomic Unit Consensus Classifier; ITS: internal transcribed spacer; NCBI: National Center for Biotechnology Information; OUT: operational taxonomic unit; rRNA: ribosomal RNA.

## Competing interests

The authors declare that they have no competing interests.

## Authors' contributions

SD preformed PCR calibration experiments, wrote and tested BROCC, and preformed sequence analysis. GP designed and tested the 18S_0067a_deg primer. SSM conducted *in silico *test analysis of the 18S_0067a_deg primer. KB participated in the development of BROCC software and preformed the synthetic community experiment and data set analysis. RS wrote BROCC's database interfaces. CH developed the PSP extraction protocol and vetted the ITS and 18S rRNA gene primers. CN, DH, and DA preformed the plasmid quantification experiment. MB generated the clinical samples. RCA extracted the clinical isolates and performed preliminary work with the 18S and ITS rRNA gene primer sets. SG extracted the human samples and worked up the human and cultured samples for sequencing. GW, JL, and FB designed the study. SD and FB wrote the paper. All authors have read and approved this manuscript for publication.

## Supplementary Material

Additional file 1 **Samples studied from known eukaryotic organisms**. Click here for file

Additional file 2 **Comparison of PCR amplification reactions for DNA purified from stool using different methods**. Average DNA yields were: PSP, 59.6 ng/μl; PowerSoil, 30.4 ng/μl; FastDNA extraction, 15.8 ng/μl; and the archaeal method, 12.7 ng/μl. PCR products were separated on an 0.8% agarose gel and stained with ethidium bromide. Top: amplification products generated using the 18S primer pair. Bottom: amplification products generated using the ITS1F-ITS2 primer pair. Click here for file

Additional file 3 **Samples studied from human stool**. Click here for file

Additional file 4 **Analysis of DNA samples from known eukaryotes using BROCC, MARTA, and MEGAN**. **(a) **18S rRNA gene amplicons classified by all three classifiers. **(b) **ITS rRNA gene amplicons classified by all three classifiers. The sample tested is listed along the x-axis. Individual OTUs in each sample are shown by the points, which are sized in proportion to their read counts. A point is colored by the program and configuration used to classify that point. These data were classified by BROCC using default settings, MARTA using default settings, MARTA using a BLAST word size and voting thresholds to match the BROCC default settings, MEGAN using default settings and the same blastn output used by BROCC, and MEGAN using an abbreviated blastn output with a maximum of five hits per query sequence. The lowest level of correct classification for each OTU is listed on the y-axis. Click here for file

Additional file 5 **Comparison of BROCC, MARTA and MEGAN**. Click here for file

Additional file 6 **Sequences of DNA oligonucleotides used in this study**. Click here for file

Additional file 7 **Description of the BROCC program**. **(a) **Pseudocode. **(b) **Flow chart of implementation. Click here for file

Additional file 8 **BROCC program parameters and options**. Defaults were used in this study. Click here for file

Additional file 9 **BROCC source code**. BROCC source code version 1.1.0. Click here for file
