# Supplementary material for: A tool kit for quantifying eukaryotic rRNA gene sequences from human microbiome samples
Source: Genome Biol. 2012 Jul 3;13(7):R60. doi: 10.1186/gb-2012-13-7-r60 (PMC4053730; doi:10.1186/gb-2012-13-7-r60)
Supplement: Additional file 1 — Samples studied from known eukaryotic organisms. [file gb-2012-13-7-r60-S1.PDF]

Samples studied from known Eukaryotic organisms.

Species	Phylum	Kingdom	Source	Euk:18s Target	Level of Classificatio	Sequence Count (18	Barcode (18s)	ITS1F/2 Target	Level of Classificatior	Sequence (	Barcode
Arabidopsis thaliana	Angiospermae	Plant	Lab Strain	No	Phylum	2485	ACGGTCGTATTG	No	Domain (Chaetospha	1708	AGCCATACTGAC
Aspergillus flavus	Ascomycota	Fungi	Clinical Isolate	Yes	Genus	2815	AACATCCTAGCG	Yes	Genus	2079	ACCGTAATCCAG
Aspergillus fumigatus M1	Ascomycota	Fungi	Clinical Isolate	Yes	Genus	2681	CAGTTGGTCTGA	Yes	Species	1465	GCTGGTATCTGA
Aspergillus fumigatus M2	Ascomycota	Fungi	Clinical Isolate	Yes	Genus	2324	TGGATATGCGCT	Yes	Species	1444	CGATAACATGCC
Aspergillus fumigatus M3	Ascomycota	Fungi	Clinical Isolate	Yes	Genus	2741	GTATTGACGGTC	Yes	Species	1697	AAGAACGTCTCC
Aspergillus niger M4	Ascomycota	Fungi	Clinical Isolate	Yes	Genus	2248	GCCGAACACTAT	Yes	Genus	1357	ACATTCAGCGCA
Aspergillus (species unknown)	Ascomycota	Fungi	Clinical Isolate	Yes	Genus	2233	TATGGCACACAC	Yes	Genus	2451	CCAATGGAACTC
Candida albicans	Ascomycota	Fungi	Clinical Isolate	Yes	Genus	2452	GCATCGTCAACA	Yes	Species	2779	GTCTGACAGTTG
Candida glabrata	Ascomycota	Fungi	Clinical Isolate	Yes	Family (Saccharomy	2877	TTGTCTGGAAGC	Yes	Species	816	TTCCGGTATGGA
Candida krusei	Ascomycota	Fungi	Clinical Isolate	Yes	Genus	2503	TCAGGACTGTGT	Yes	Species	2710	GTCGTGTGTCAA
Candida parapsilosis	Ascomycota	Fungi	Clinical Isolate	Yes	Genus	2879	TTAGGTGCAGCT	Yes	Species	1749	CGAACTCAATGC
Candida tropicalis	Ascomycota	Fungi	Clinical Isolate	Yes	Genus	2450	GTCTCATGTAGG	Yes	Species	1779	TCCAAGTAAGCC
Coccidioides immitis	Ascomycota	Fungi	Lab Strain	Yes	Class	2614	GTTCGCGTATAG	Yes	Genus	1585	ACCTCGATCAGA
Cryptococcus laurentii	Basidiomycota	Fungi	Clinical Isolate	Yes	Phylum^	2435	TATCAGGTGTGC	Yes	Species	2669	CTAGCGAACATC
Crytpococcus neoformans	Basidiomycota	Fungi	Clinical Isolate	Yes	Species	2525	ATCGATCTGTGG	Yes	Species	2630	CTGGCTGTATGA
Dematiaceus fungi spp.	Ascomycota	Fungi	Clinical Isolate	Yes	Phylum†	2288	GTATGACTGGCT	Yes	Genus†	1789	TGAGGTCTTGAC
Human 293T	Chordata	Animal	Cell Line	No	Domain (Penicillilum	13	GTGACCTGATGT	No	Domain (Phaeosphae	347	TGTCAAGTCGTG
Leishmania mexicana	Euglenozoa	Excavate	Lab Strain	Yes	Family	2117	TCAGCCATGACA	No	Domain (Pichia)	48	TCGACCAGCAAT
Penicillium spp.	Ascomycota	Fungi	Clinical Isolate	Yes	Genus	2657	TAATCCACAGCG	Yes	Genus	1146	CGTCTCAGAACA
Plasmodium falciparum 7G8	Apicomplexa	Chromalveolate	Lab Strain	No	Domain	41	GTCAAGAACCTC	No	Domain (Penicillium)	28	ACGTTAGCACAC
Plasmodium falciparum TG94	Apicomplexa	Chromalveolate	Lab Strain	No	Domain (Candida)	2209	TTGCACGATTGG	No	Domain (C. parapsilo	1763	CATGTTGGCATG
Pneumocystis	Ascomycota	Fungi	Mouse Lung Isolate	Yes	Genus	2495	GTAGAGCTGTTC	Yes	Genus*	495	TCACAGATCCGA
Saccharomyces cerevisiae	Ascomycota	Fungi	Environmental Isolate	Yes	Genus	1891	TAGTTGCGAGTC	Yes	Species	641	GACTAACGTCAC
Toxoplasma gondi	Apicomplexa	Chromalveolate	Lab Strain	Yes	Family	2725	GACCACTACGAT	No	Domain (Tremellomy	84	CCTATGTGATGG

*Sample not typed to species level, but classifier made an assignment

†Sample not typed such that it is consistant with taxonomy, typer suspected Exophiala jeanselmei

^Sample reclassified correctly to the family level after list of generic database classifications appended
