# Supplementary figures and images for: A tool kit for quantifying eukaryotic rRNA gene sequences from human microbiome samples
Source: Genome Biol. 2012 Jul 3;13(7):R60. doi: 10.1186/gb-2012-13-7-r60 (PMC4053730; doi:10.1186/gb-2012-13-7-r60)

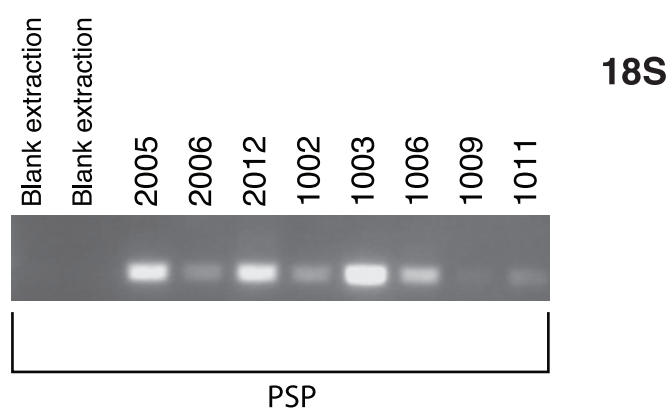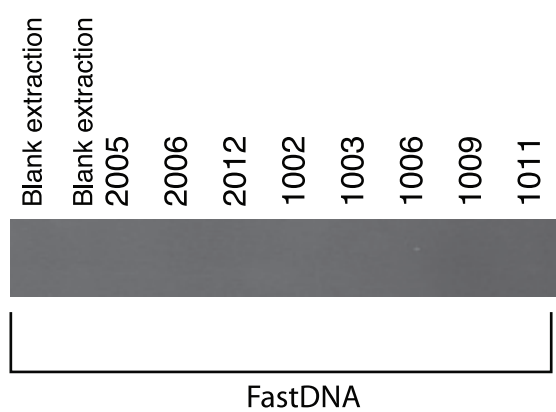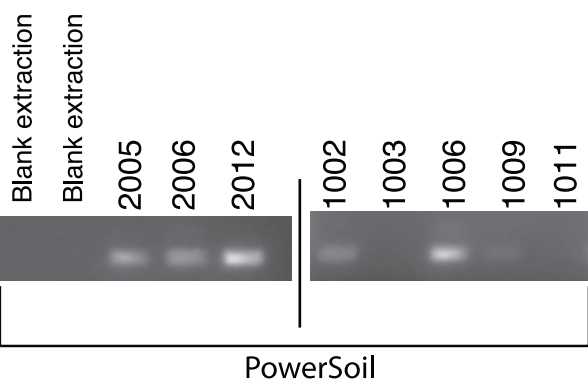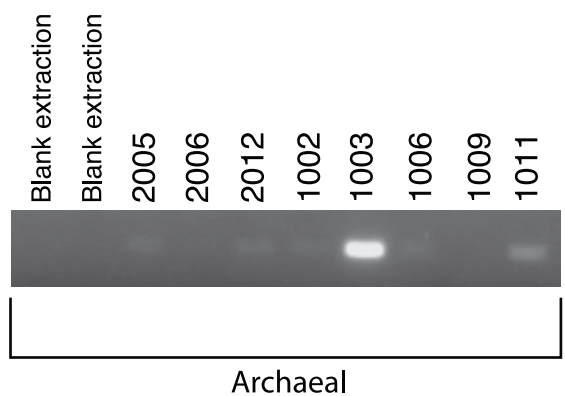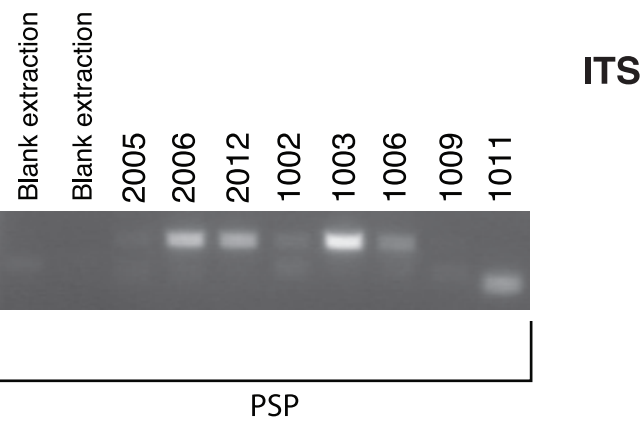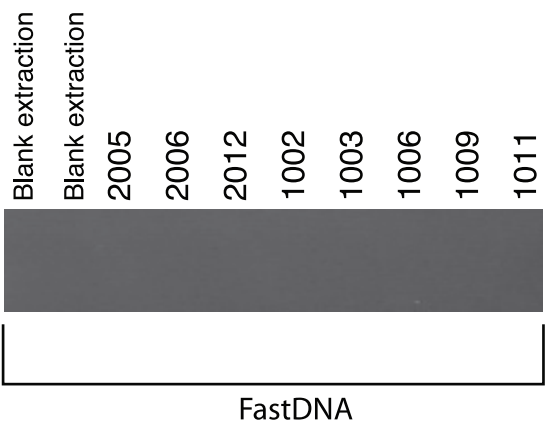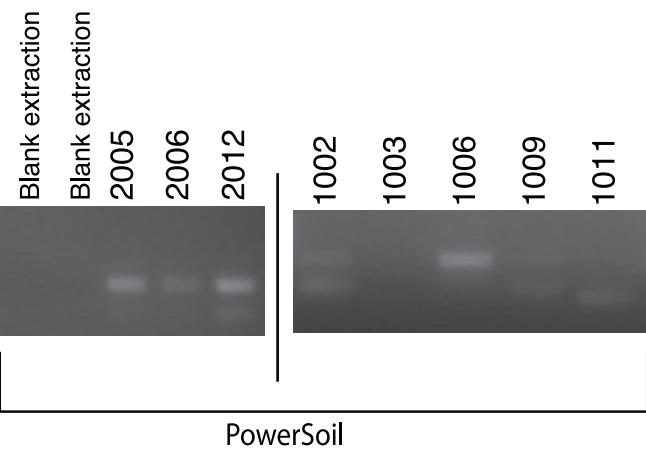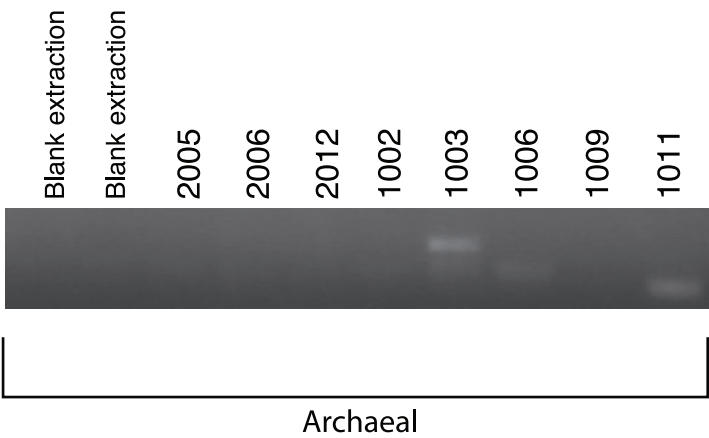

Supplement: Additional file 2 — Comparison of PCR amplification reactions for DNA purified from stool using different methods. Average DNA yields were: PSP, 59.6 ng/μl; PowerSoil, 30.4 ng/μl; FastDNA extraction, 15.8 ng/μl; and the archaeal method, 12.7 ng/μl. PCR products were separated on an 0.8% agarose gel and stained with ethidium bromide. Top: amplification products generated using the 18S primer pair. Bottom: amplification products generated using the ITS1F-ITS2 primer pair. [file gb-2012-13-7-r60-S2.PDF]

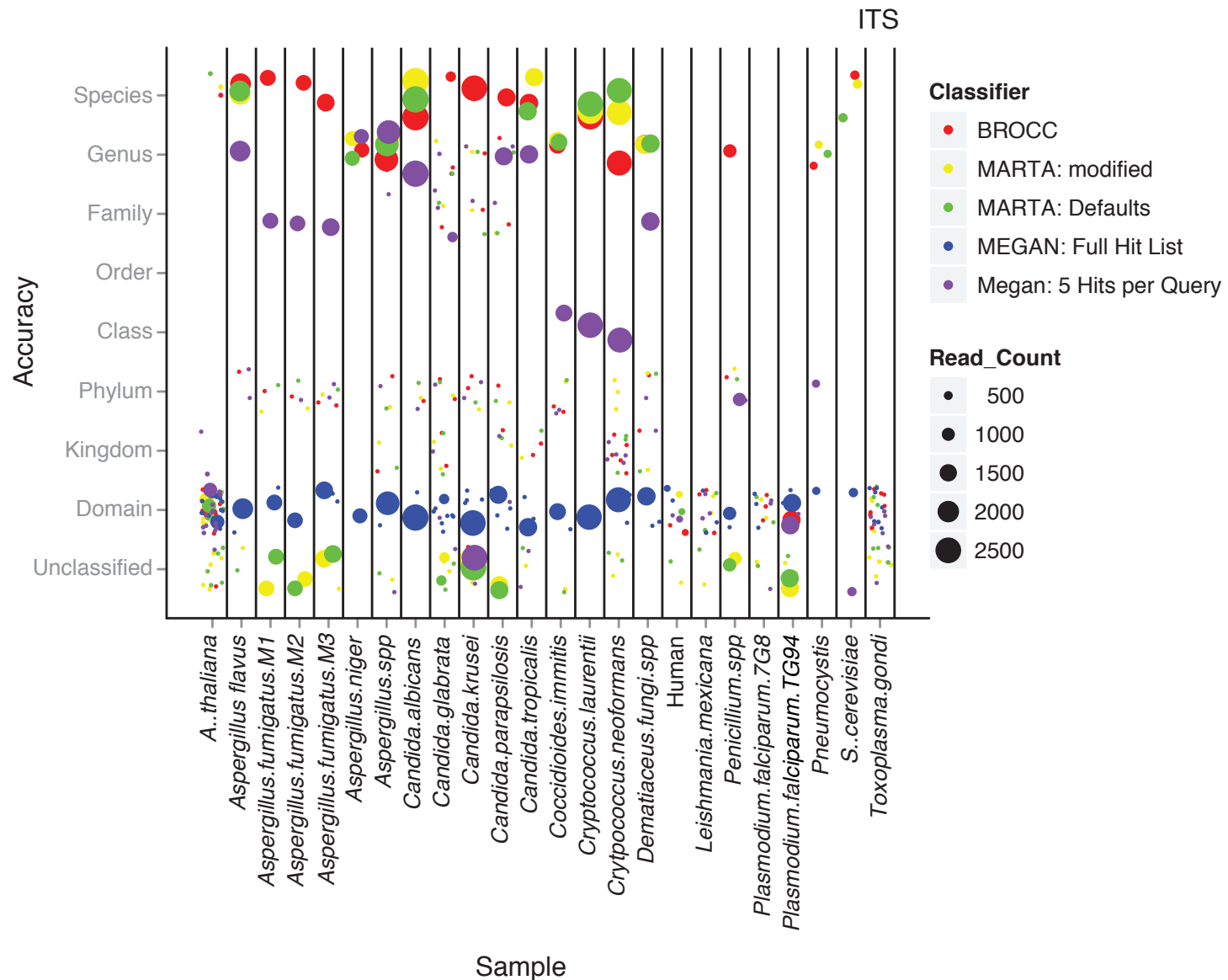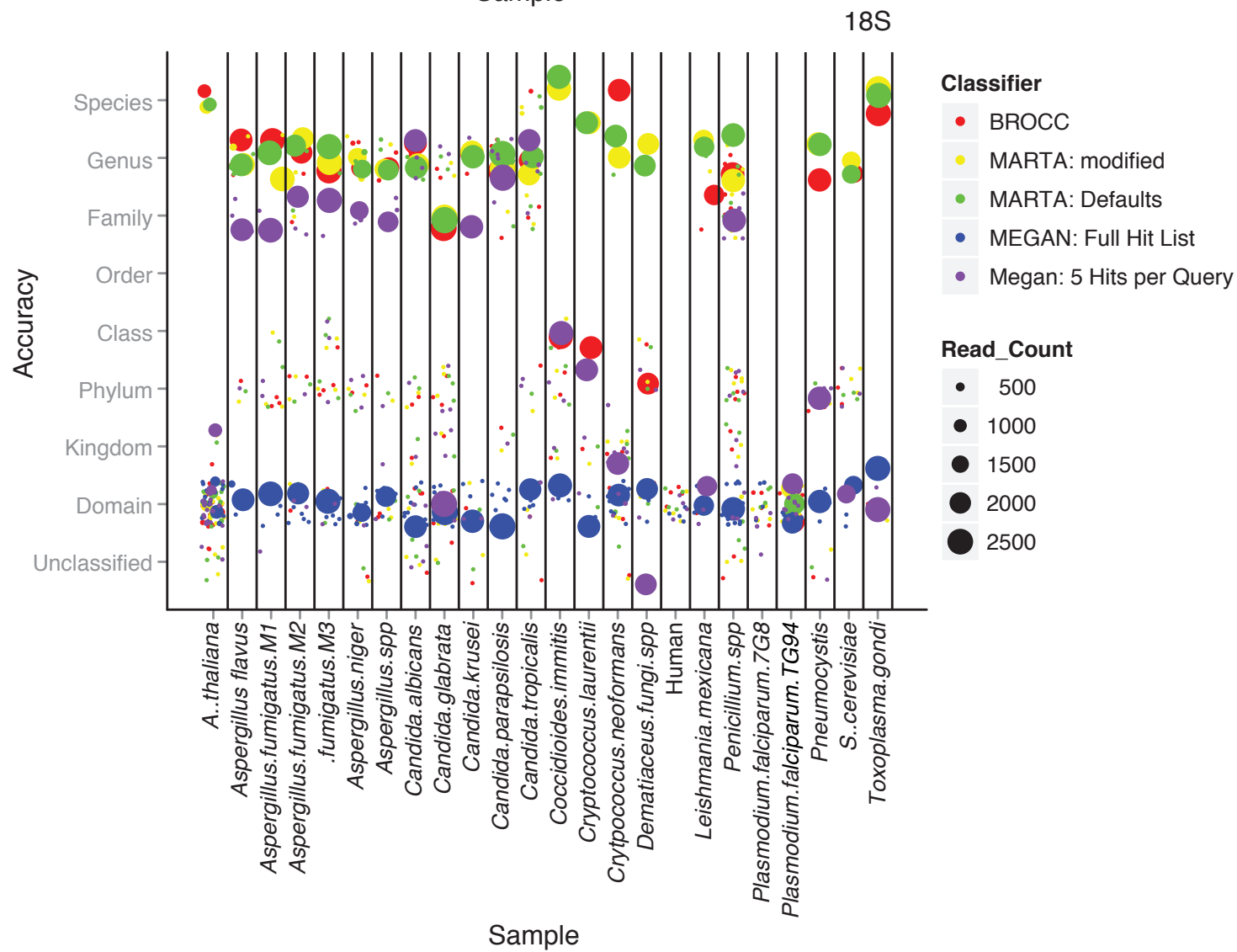

Supplement: Additional file 4 — Analysis of DNA samples from known eukaryotes using BROCC, MARTA, and MEGAN. (a) 18S rRNA gene amplicons classified by all three classifiers. (b) ITS rRNA gene amplicons classified by all three classifiers. The sample tested is listed along the x-axis. Individual OTUs in each sample are shown by the points, which are sized in proportion to their read counts. A point is colored by the program and configuration used to classify that point. These data were classified by BROCC using default settings, MARTA using default settings, MARTA using a BLAST word size and voting thresholds to match the BROCC default settings, MEGAN using default settings and the same blastn output used by BROCC, and MEGAN using an abbreviated blastn output with a maximum of five hits per query sequence. The lowest level of correct classification for each OTU is listed on the y-axis. [file gb-2012-13-7-r60-S4.PDF]
