# Supplementary material for: A tool kit for quantifying eukaryotic rRNA gene sequences from human microbiome samples
Source: Genome Biol. 2012 Jul 3;13(7):R60. doi: 10.1186/gb-2012-13-7-r60 (PMC4053730; doi:10.1186/gb-2012-13-7-r60)
Supplement: Additional file 3 — Samples studied from human stool. [file gb-2012-13-7-r60-S3.PDF]

Samples of human stool studied.

			18s								
				Archaeal Extraction			PowerSoil			PSP	
Sample Name	Species	Disease State		Sequence C	Barcode		Sequence C	Barcode		Sequence C	Barcode
1002	Human	Healthy	Archaeal2.FSM.10(	384	ATGAGACTCCAC	PS.FSM.1002	3506	TAATCCACA	PSP.FSM.1002	3309	TCGAATCACAGC
1003	Human	Healthy	Archaeal2.FSM.10(	3920	GTGTTGCAGCAT	PS.FSM.1003	94	GTAGAGCT	PSP.FSM.1003	4432	AGTGTGGACTTC
1006	Human	Healthy	Archaeal2.FSM.10(	565	TGCGTCAGTTAG	PS.FSM.1006	2879	TAGTTGCG	PSP.FSM.1006	3454	CTCCACATGAGA
1009	Human	Healthy	Archaeal2.FSM.10(	0	ATGTTGGCTACG	PS.FSM.1009	2139	TATCAGGT(	PSP.FSM.1009	1899	TGTGGTACTACG
1011	Human	Healthy	Archaeal2.FSM.10:	3441	TGCTCTAGTGGA	PS.FSM.1011	124	ATCGATCTG	PSP.FSM.1011	3314	CACATGCCTAAG
2005	Human	Healthy	Archaeal2.Caf..200	709	GTCAAGAACCTC	PS.Caf..2005	3473	GTCTCATGT	PSP.Caf..2005	1955	CTTCGTGGTAGA
2006	Human	Healthy	Archaeal2.Caf..200	174	TTGCACGATTGG	PS.Caf..2006	3821	GTTCGCGT	PSP.Caf..2006	3574	TAGGATTGCTCG
2012	Human	Healthy	Archaeal2.Caf..201	725	GACCACTACGAT	PS.Caf..2012	3833	GTATGACT(	PSP.Caf..2012	2899	AGCGCAACATTC
Neg 1			Archaeal2.H2O.1	3	GTGACCTGATGT	PS.H2O.1	15	TCAGGACT	PSP.H20.1	243	GCACACACGTTA
Neg 2			Archaeal2.H20.2	50	TCAGCCATGACA	PS.H2O.2	15	TTAGGTGC	PSP.H20.2	272	TTCCTAGGTGAG
Extraction Subtotal				9971			19899			25351	
					Amplicon Total	55221					

	ITS								
		Archaeal Extraction		PowerSoil			PSP	
Sample Name		Sequence C	Barcode	Sequence C	Barcode		Sequence C	Barcode
1002	Archaeal2.FSM.1002	480	GGCTTGTACTAG	PS.FSM.10(	2649	PSP.FSM.10	1358	CGAGTCTAGTTG
1003	Archaeal2.FSM.1003	2030	CGGACTACAACT	PS.FSM.10(	0	PSP.FSM.10	1000	TGACAATCCAGC
1006	Archaeal2.FSM.1006	3064	TATGGATTCCGG	PS.FSM.10(	1217	PSP.FSM.10	2098	ACCGTAATCCAG
1009	Archaeal2.FSM.1009	1	CACCAGATTCAG	PS.FSM.10(	2581	PSP.FSM.10	2005	GCTGGTATCTGA
1011	Archaeal2.FSM.1011	4349	AGTTAGTGCGTC	PS.FSM.101	3630	PSP.FSM.10	3568	CGATAACATGCC
2005	Archaeal2.Caf..2005	433	CTATACCACGGA	PS.Caf..200	1189	PSP.Caf..20(	1775	CTTGACTGAGGT
2006	Archaeal2.Caf..2006	510	GTATAGGTTCGC	PS.Caf..200	1120	PSP.Caf..20(	1096	AGCTATCCACGA
2012	Archaeal2.Caf..2012	129	AGTGTTCGATCG	PS.Caf..201	1076	PSP.Caf..20:	1278	GAACTAGTCACC
Neg 1	Archaeal2.H2O.1	168	CTGTGTTCAGGA	PS.H2O.1	121	PSP.H20.1	1551	GTCACCGAACTA
Neg 2	Archaeal2.H2O.2	394	ACGCAACTGCTA	PS.H2O.2	180	PSP.H20.2	170	TGCGCTTGGATA
Extraction Subtotal		11558			13763		15899	
			Amplicon T	41220				
