# Supplementary material for: A tool kit for quantifying eukaryotic rRNA gene sequences from human microbiome samples
Source: Genome Biol. 2012 Jul 3;13(7):R60. doi: 10.1186/gb-2012-13-7-r60 (PMC4053730; doi:10.1186/gb-2012-13-7-r60)
Supplement: Additional file 5 — Comparison of BROCC, MARTA and MEGAN. [file gb-2012-13-7-r60-S5.PDF]

Comparison of BROCC, MARTA, and MEGAN.

BROCC	MARTA	MEGAN
Votes on multiple high identity % identities	Votes within highest bit score	Selects Lowest Common Ancestor above bit score threshold
Uses percent identity	Uses bitscore	Uses bit score
Filters for percent identity, coverage, and classification level	Filters for coverage and percent identity	Filters for bitscore
Adjustable consensus thresholds at each taxonomic rank	Adjustable consensus thresholds at each taxonomic rank	Requires full consensus at assigned rank
Adjustable coverage filter	80% coverage filter	No filter
Excludes adjustable set of generic db classifications	Excludes all "uncultured" or "unidentified" db classifications	Includes generic db hits in decision
Begins voting at bottom of taxonomy	Begins voting at bottom of taxonomy	n/a
Can use any blast implementation	Uses megablast	Can use any blast implementation
Queries NCBI online for taxonomy	Queries SQL database for taxonomy	Uses NCBI taxonomy in program
Implemented in Python	Implemented in Java	Implemented in Java
Can integrate into Qiime	Requires intervention for use with Qiime	Can integrate into Qiime
