# Supplementary material for: A tool kit for quantifying eukaryotic rRNA gene sequences from human microbiome samples
Source: Genome Biol. 2012 Jul 3;13(7):R60. doi: 10.1186/gb-2012-13-7-r60 (PMC4053730; doi:10.1186/gb-2012-13-7-r60)
Supplement: Additional file 6 — Sequences of DNA oligonucleotides used in this study. [file gb-2012-13-7-r60-S6.PDF]

Sequences of DNA oligonucleotides used in this study.

Primer	Amplicon	Sequence
18S_0067a_deg	18s	AAGCCATGCATGYCTAAGTATMA
NSR 399	18s	TCTCAGGCTCCYTCTCCGG
ITS1F	ITS1	CTTGGTCATTTAGAGGAAGTAA
ITS2	ITS1	GCTGCGTTCTTCATCGATGC
