# Supplementary material for: A tool kit for quantifying eukaryotic rRNA gene sequences from human microbiome samples
Source: Genome Biol. 2012 Jul 3;13(7):R60. doi: 10.1186/gb-2012-13-7-r60 (PMC4053730; doi:10.1186/gb-2012-13-7-r60)

A

INPUT:

H, array of BLAST-like database hits
c, minimum coverage threshold
I, array of minimum percent identity thresholds
P, array of minimum consensus thresholds

FOR each taxonomic rank, r, beginning with species:
LET V be an array of votes
FOR each taxon, i, in rank r:
$V_i = 0$
FOR each BLAST-like database hit:
NEXT hit if coverage < c
NEXT hit if percent identity < I_r
m <- taxon assignment for hit at rank r
INCREMENT V_m
k <- argmax(V)
IF (V_k / sum(V)) > P_r
OUTPUT r, k

B

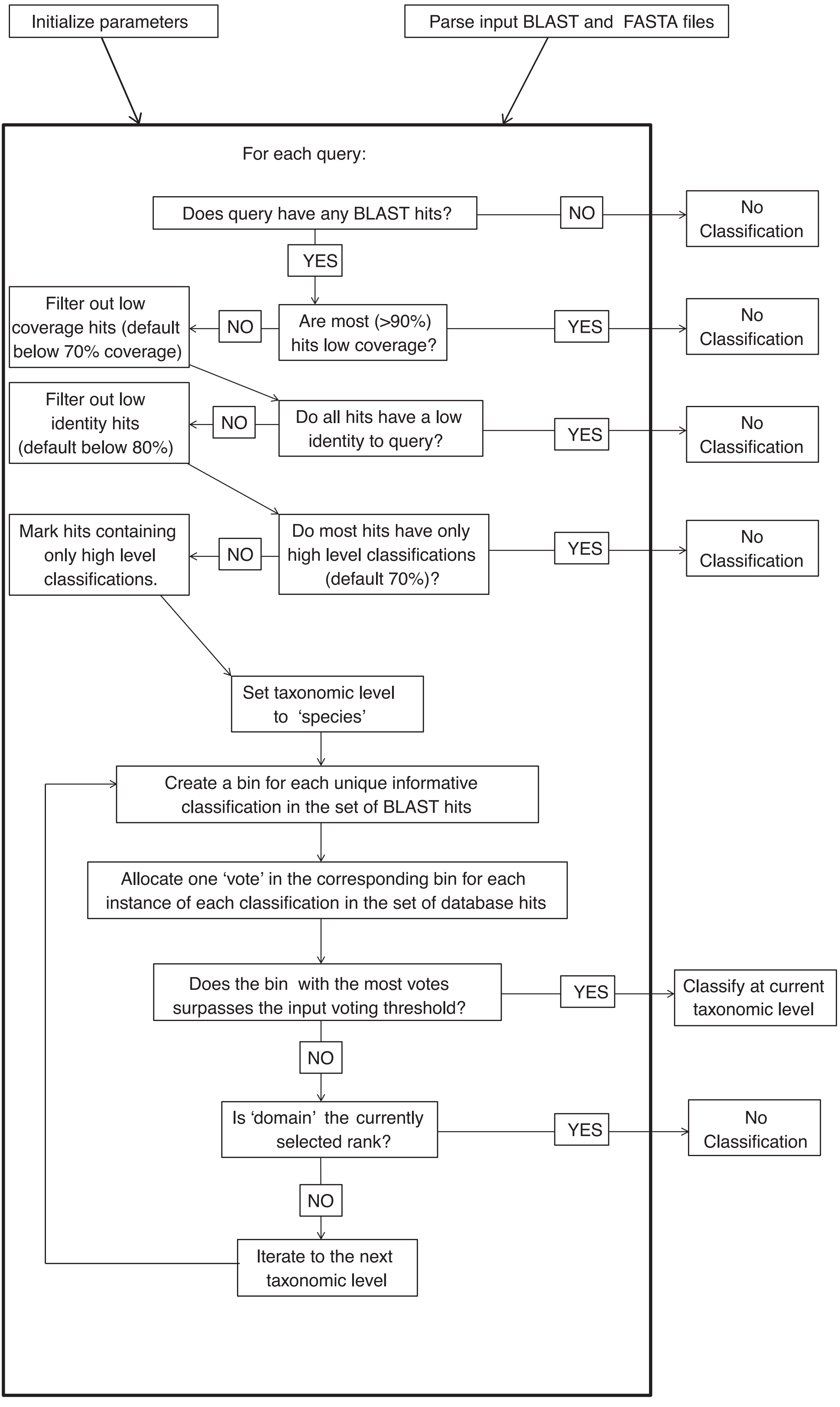

Supplement: Additional file 7 — Description of the BROCC program. (a) Pseudocode. (b) Flow chart of implementation. [file gb-2012-13-7-r60-S7.PDF]
