# Supplementary material for: A tool kit for quantifying eukaryotic rRNA gene sequences from human microbiome samples
Source: Genome Biol. 2012 Jul 3;13(7):R60. doi: 10.1186/gb-2012-13-7-r60 (PMC4053730; doi:10.1186/gb-2012-13-7-r60)
Supplement: Additional file 8 — BROCC program parameters and options. Defaults were used in this study. [file gb-2012-13-7-r60-S8.PDF]

BROCC parameters used in this study.

Option Type	Option	Function	Accepts	Default
Logistic	-h, --help	Displays all possible options	n/a	
Logistic	-v	Output to console when each query is classified	n/a	Off
Logistic	-q	Run BROCC quietly	n/a	On
Filter Parameter	--min_cover	Minimum coverage of query sequence required for a database hit to be considered for classification	Decimal	70%
Filter Parameter	--min_id	Minimum identity required for a database hit to be considered for classification above the genus level	Percentage	80%
Filter Parameter	--min_genus_id	Minimum identity required for a database hit to be considered at the genus level	Percentage	
Filter Parameter	--min_species_id	Minimum identity required for a database hit to be considered at the species level	Percentage	
Filter Parameter	-a, --amplicon	Predefined species and genus identity settings for 18S rDNA (.99, .96) and ITS1 (.952,.8305) Amplicons	The strings 'ITS' or '18S'	
Filter Parameter	--max_generic	Maximum number of generic classifications for database hits allowed before query cannot be classified	Decimal	70%
Input/Output	-c, --config	Path to configuration file	.cfg file provided by authors, editable by user	
Input/Output	-i, --input_fasta_file	Path to FASTA file of query sequences	FASTA file	
Input/Output	-b, --input_blast_file	Path to BLAST file	BLAST output format 7 file	
Input/Output	-o, --output_directo	Path to output folder	File path existing folder or folder for BROCC to create	
